# Lord Knutsford's "Impossible."

**Published:** 1919-12-20

**Authors:** 


### LORD KNUTSFORD'S "IMPOSSIBLE."

When Lord Knutsford declares that a forty-eight-hour week for nurses is impossible the outstanding fact that presents itself to a mind like mine is that there is no hope in this quarter. The author of the Nurses' Charter of Liberty is pretty well where he was when he advised nurses not to stand it if they were not allowed as a minimum two hours' liberty in twenty-four. I do not seek to pillory the Chairman of the "London" for his sentiments. Far be the thought. I repeat what I stated last week—that I admire Lord Knutsford for his outspokenness. There are others who share the same views, but who do not express them.

These people, it appears to me, do not take the hospital nurse seriously. They flatter her. I am always suspicious of flattery. It usually signifies cheap remuneration. I see no reason why a nurse should be praised for nursing if it is her business, but I see a score of reasons why she should be paid. If this view is not sound I should like to see the case rebutted.

Now I want to tell Lord Knutsford a little story. Probably he never heard it before, and, even if he did, it will bear repetition.

Just a year before the war, that is to say in the year 1913, the State Bureau of Labour in California introduced a Bill to the Legislature including hospitals amongst other institutions and employers of women who already enjoyed an eight-hour day. This Bill created terrible consternation, and was vigorously opposed. It did not apply to trained nurses, but to probationers only. I am indebted for my information to an article by Anna C. Jamme, R.N., in the *American Journal of Nursing* for April of this year. Nurse Jamme points out that the complainants attacked the Act on the ground that it interfered with liberty of contract. I remember when I attacked Lord Knutsford's Charter of Liberty his answer was that the probationer made a bargain, and ought to keep it. It is remarkable how hospital probationers and hospital Committees think on the same respective lines in London and in California. The writer of the article states that "the opposition was represented by the owners of hospitals, trustees, and superintendents." If a Bill were introduced to Parliament to-morrow to accomplish the same thing in England I can easily imagine history repeating itself. "The ground taken," says Nurse Jamme, "was that students were not in industry, but in a school; that a larger number of students would be required, and consequently there would be greater expense to hospitals"; and so forth. But the greatest objection, apparently, was that "disciplinary standards would suffer, owing to the student having too much leisure time."

Fancy all this array of organised opposition. But this was not all. The physicians joined the procession, and objected to the frequent changes of nurses, and the inability to fix responsibility on any one nurse. One hospital went so far as to attack the constitutionality of the law, and carried it to the Supreme Court of the United States. The end of the story is that the State of California triumphed, the probationer triumphed, the Supreme Court of the United States holding that "the propriety of legislative protection of women undergoing such a discipline is not open to question." The Act came into operation in August 1913, and the author of the article says that "the practical working out of the law during its five years of existence has shown it to be, in the first place, a possibility, even with the inflexibility of the forty-eight hour week. It has taken time to bring it into smooth running order and into favour. At present many superintendents who were strongly opposed at first now express satisfaction and unwillingness to go back to the former plan, even should the law be repealed." She also states that on August 1, when the law became effective, superintendents of nurses had their schedules arranged, and every hospital had the plan ready to inaugurate. "It was law, and had to be done!"

Now I think this story extremely interesting. And it all happened before the war, before the British nation discovered and realised the intrinsic value of the nurse, and the intrinsic value of her services to the nation. And hence, when people like Lord Knutsford tell us that an eight-hour day and a forty-eight-hour week are "impossible," I think I am justified in entering a protest.

IERNE.

